# Effectiveness of chlorhexidine gels and chips in Periodontitis Patients after Scaling and Root Planing: a systematic review and Meta-analysis

**DOI:** 10.1186/s12903-023-03241-2

**Published:** 2023-10-29

**Authors:** Zahratul Umami Annisa, Benso Sulijaya, Ette Soraya Shahnaz Tadjoedin, Dimas Ilham Hutomo, Sri Lelyati C. Masulili

**Affiliations:** 1 Undergraduate Program, Faculty of Dentistry, Universitas Indonesia, Jl. Salemba Raya No. 4, 10430 Jakarta Pusat, Indonesia; 2 Department of Periodontology, Faculty of Dentistry, Universitas Indonesia, Jl. Salemba Raya No. 4, 10430 Jakarta Pusat, Indonesia

**Keywords:** Chlorhexidine chip and gel, Metronidazole gel, Minocycline microspheres, Scaling and root planing, Tetracycline fibers

## Abstract

Periodontal pockets are characteristic of periodontitis. Scaling and root planing is the gold standard for periodontitis treatment. Additional local antimicrobials are recommended in patients with a probing depth of ≥ 5 mm. This study aims to determine the effectiveness of chlorhexidine compared to other local antimicrobials in periodontitis. Searches were conducted using the Preferred Reporting Items for Systematic Reviews and Meta Analysis (PRISMA) guidelines. Meta-analysis was performed on studies that met inclusion criteria after risk of bias assessment. Meta-analysis between chlorhexidine chips and other antimicrobials showed a mean difference in probing depth after one month of 0.58 mm (p < 0.00001) whereas after three months the mean difference in probing depth was 0.50 mm (p = 0.001), index plaque 0.01 (p = 0.94) and gingival index − 0.11 mm (p = 0.02). Between chlorhexidine gel and other antimicrobials showed a mean difference in probing depth of 0.40 mm (p = 0.30), plaque index of 0.20 mm (p = 0.0008) and gingival index of -0.04 mm (p = 0.83) after one month. Chlorhexidine chips were more effective on the gingival index than other antimicrobials after three months. The other antimicrobials were more effective than chlorhexidine chips on probing depth after one and three months, and than chlorhexidine gels on plaque index after one month.

## Introduction

Periodontal disease is one of the main causes of tooth loss which affects the masticatory ability, aesthetics, self-confidence, and quality of life of individuals [1, 2]. Based on the Indonesian National Basic Health Research (RISKESDAS) 2018, 7 out of 10 people in Indonesia experience periodontitis [3]. The prevalence of periodontal disease is expected to continue to increase in the coming years [2]. The pathogenesis of periodontal disease is initiated by a group of microorganisms that will modulate the host response by interfering with the immune response and changing the balance from homeostasis to dysbiosis [4].

Mechanical debridement in the form of scaling and root planning is considered the gold standard non-surgical procedure for periodontal therapy [5]. Scaling and root planing aims to remove plaque biofilm, calculus, and endotoxin from the tooth surface. Scaling and root planing have limitations and their impact on some patients or under certain conditions is not optimal [6, 7]. Scaling and root planing may fail because of limited access of periodontal instruments [7]. When the depth of the periodontal pocket becomes 5 mm or more, scaling and root planning becomes less effective. Additional antimicrobials are proposed to overcome these problems [8].

Antimicrobials as adjunctive therapy after scaling and root planing can be used systemically or locally [9]. Various studies revealed that local antimicrobials in the periodontal pocket can provide higher therapeutic concentrations of antibiotics compared to systemic administration [10]. Periodontitis is a localized disease, therefore local treatment is preferable to systemic therapy to avoid complications associated with systemic antimicrobial administration [10]. The most common local antimicrobials used as local antimicrobials in the treatment of periodontitis are chlorhexidine, minocycline, metronidazole, and tetracycline [11].

Chlorhexidine is a broad-spectrum antibacterial agent that is effective in treating periodontal disease [12]. The concentration of chlorhexidine varies depending on the preparation used, namely chlorhexidine chips and chlorhexidine gel with xanthan gum [13]. The chlorhexidine chip contains 2.5 mg of chlorhexidine gluconate, incorporated in the matrix [14]. Ma, et al. performed a meta-analysis and found that scaling and root planing with the addition of chlorhexidine chips showed better clinical outcomes than scaling and root planing alone in patients with periodontitis [15]. The chlorhexidine gel contains 1.5% chlorhexidine in a xanthan gum matrix [14]. Chlorhexidine gel can be used as an adjunct for scaling and root planing and is more effective than scaling and root planing alone in the treatment of periodontitis [16]. The use of chlorhexidine has long-term side effects, such as extrinsic tooth staining and calculus formation [17, 18]. Previous systematic reviews have demonstrated significant beneficial effects on scaling and root planing treatments with additional local antimicrobials, such as chlorhexidine chips and gels, monocycline microspheres, metronidazole gel, and tetracycline fibers compared to scaling and root planing alone in patients with periodontitis [13, 19, 20].

The effectiveness of local antimicrobial use can be assessed at one and three months after treatment. It was estimated that the chlorhexidine chip and gel would be completely adsorbed after 30 days of placement in the periodontal pocket [21]. The bacteria are expected to return to their pre-treatment pattern three to six weeks after scaling and root planing [22]. The three-month timeframe corresponds to the control interval for periodontitis patients [12]. We hypothesize that adjunctive therapy of antimicrobials in specific delivery system may improve periodontal parameters. Comparison of effectiveness between local antimicrobials is currently unclear and there has not been a systematic review of the effectiveness comparison between chlorhexidine chips and gels with other local antimicrobials after scaling and root planing. Therefore, a systematic review and meta-analysis regarding the comparative effectiveness of chlorhexidine chips and gel with other local antimicrobials after scaling and root planing in periodontitis patients is warranted.

## Materials and methods

This research is a retrospective observational study in the form of systematic reviews and meta-analyses with the guidelines of Preferred Reporting Items for Systematic Reviews and Meta-Analysis (PRISMA) which is a statistical technique for combining the results of two or more similar studies so that a combination of qualitative and quantitative data is obtained. Research The study was conducted by tracing research that has been published in ProQuest, PubMed, EBSCO, ScienceDirect, and Scopus. The study population was an entire study that included a comparison of the effectiveness of chlorhexidine compared to other local antimicrobials (metronidazole, tetracycline, and minocycline) after scaling and root planing (SRP) in periodontitis patients. In brief, the inclusion criteria are: clinical trial studies regarding the administration of chlorhexidine with other local antimicrobials (metronidazole, tetracycline, and minocycline) after scaling and root grinding in periodontitis patients; publications of the last 10 years; full paper publication in English; studies with results in periodontal pocket depth, gingival index, and/or plaque index; studies with participant probing depth of at least 5 mm for periodontitis; and, studies with a follow-up duration of 30 days or 1 month. While the exclusion criteria are: studies in the form of systematic reviews, meta-analyses, animal studies, case series, and case reports; studies with repeated antimicrobial administration; studies with participants with systemic disease; and studies with smoking participants.

The research question in this study was “How is the effectiveness of chlorhexidine compared to other local antimicrobials after root scaling and planing in periodontitis patients?“. This research question is translated using PICO which consists of Population, Intervention, Comparison, and Outcome. A table of PICO description is provided in Table 1. From the PICO analysis, keywords can be arranged using a combination of the word’s periodontitis, scaling, root planning, chlorhexidine, tetracycline, minocycline, metronidazole, gel, and chip. The search strategy was carried out by using the words “OR” and “AND” for combinations of keywords such as “periodontitis AND scaling OR root planing AND chlorhexidine AND tetracycline OR minocycline OR metronidazole AND gel OR chip”.

**Table 1:** PICO Description

*Population (P)*	*Intervention (I)*	*Comparison (C)*	*Outcome (O)*
Periodontitis Patients	Scaling and root planing with additional local antimicrobial in the form of chlorhexidine.	Scaling and root planing with additional local antimicrobials such as metronidazole, tetracycline, and minocycline.	Primary outcome • Probing depth Secondary outcome • Gingival index • Plaque index

The review protocol is recorded in the PROSPERO database under the number CRD42022351534. Keywords consist of periodontitis, scaling, root planning, chlorhexidine, tetracycline, minocycline, metronidazole, gel, and chip. The search strategy is carried out by using the words “OR” and “AND” for a combination of keywords which are then used to access the electronic database. The study selection process was carried out using the Rayyan website. Duplication of search results from other databases will be excluded. Furthermore, inappropriate titles and abstracts will be excluded. The studies obtained will be reviewed as a whole to meet the specified inclusion and exclusion criteria which will then be assessed for the risk of bias and included in the qualitative synthesis and quantitative synthesis (meta-analysis). Processing of the data obtained will be carried out using Software Review Manager which will obtain the result in the form of an overall mean in the form of a forest plot.

## Results

### Research Identification and Selection

**Fig. 1:**
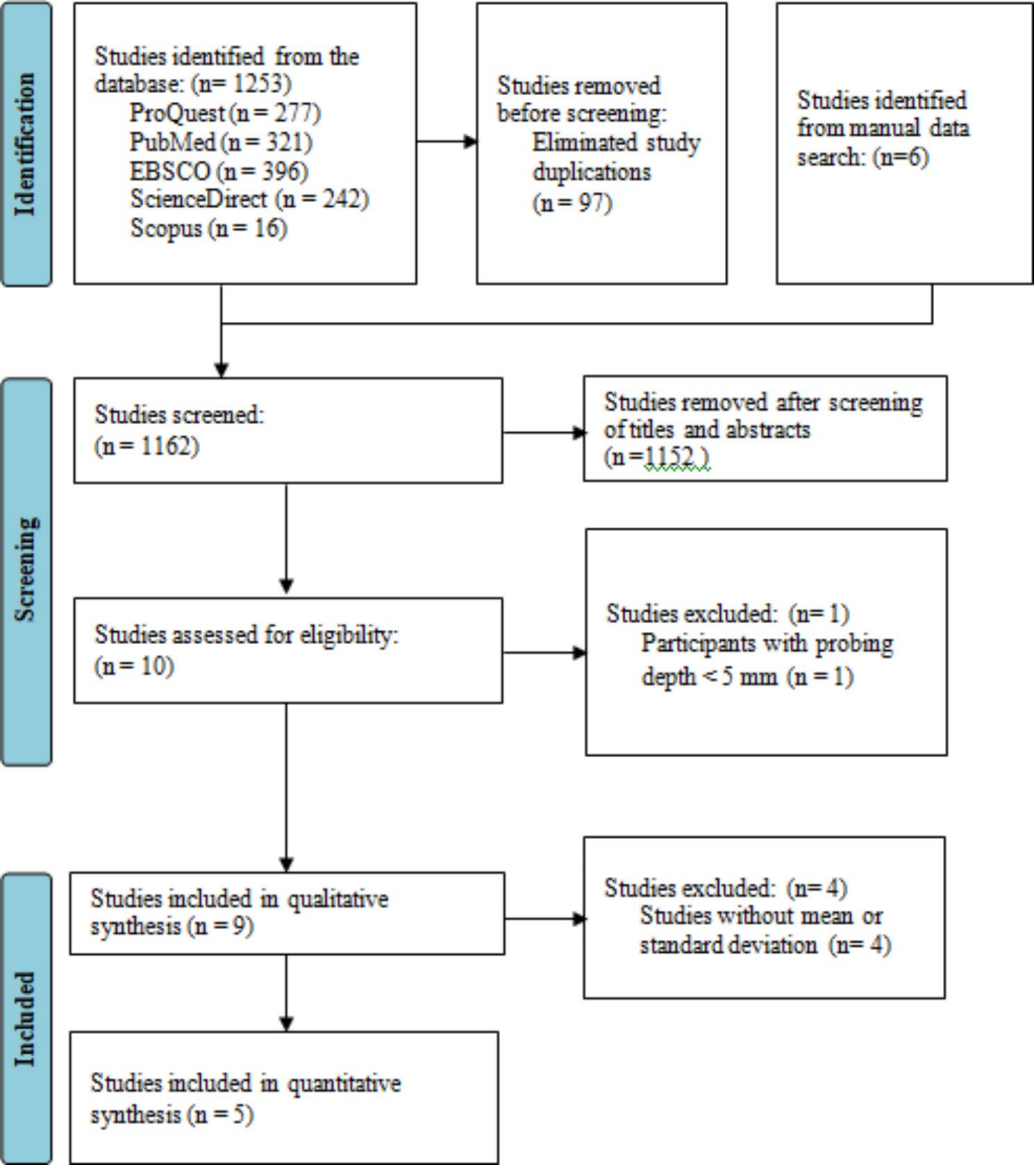
PRISMA flow diagram

Research identification was carried out according to PRISMA (Preferred Reporting Items for Systematic Reviews and Meta-Analyses) guidelines. A flow chart is provided in Fig. 1. Research identification began by searching five electronic databases, namely ProQuest, PubMed, EBSCO, ScienceDirect, and Scopus. The search was carried out using combination keywords such as periodontitis, scaling, root planing, chlorhexidine, tetracycline, minocycline, metronidazole, gel, and chip. There are nine studies for qualitative synthesis and five studies for quantitative synthesis.

The search results from the five electronic databases with these keywords yielded a total of 1253 studies, including 278 studies from ProQuest, 321 studies from Pubmed, 396 studies from EBSCO, 242 studies from ScienceDirect, and 16 studies from Scopus. The studies identified from other sources are six studies. All of these studies were deduplicated using the Rayyan website and found 97 repeated studies. Title and abstract screening was carried out in 1162 studies and 1152 studies were excluded because they did not comply with the inclusion and exclusion criteria that had been set by the authors so that the remaining 10 studies had their full text read. From the reading of the full manuscript, one study was excluded because study participants had a probing depth of < 5 mm (n = 1). There were nine studies included for the qualitative synthesis. The quantitative synthesis of four studies was excluded because the studies did not include the mean and standard deviation (n = 4) so that the quantitative synthesis was carried out in five studies.

### Risk of Bias Assessment

**Fig. 2:**
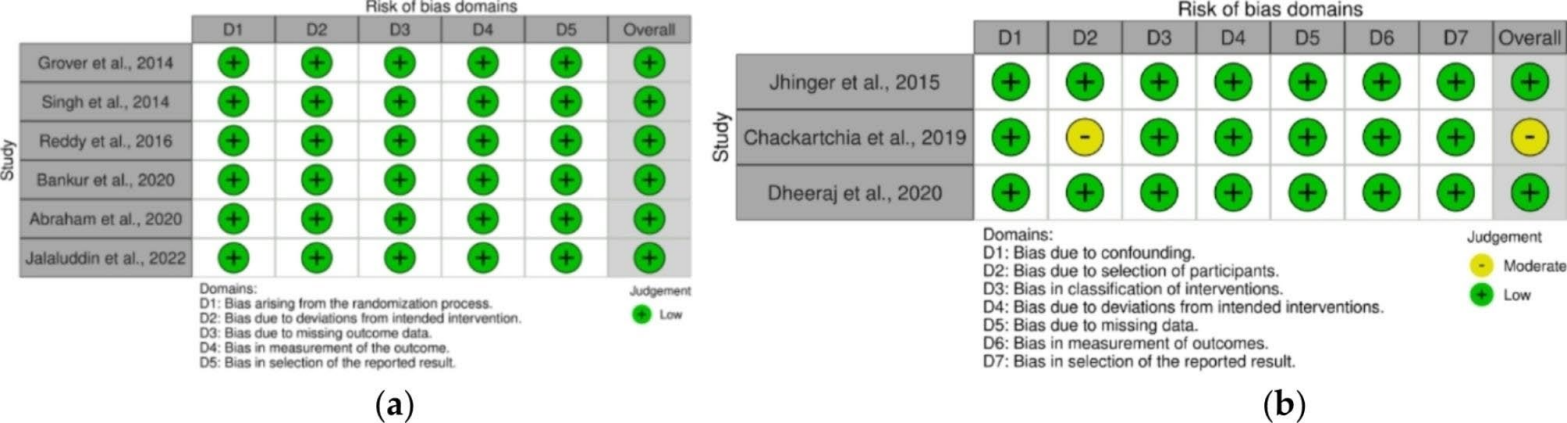
The Results of the Risk of Bias Assessment (**a**) Assessment of the Risk of Bias for Randomized Clinical Trials. (**b**) Assessment of the Risk of Bias for Non Randomized Clinical Trials

The risk of bias assessment for randomized clinical trials was carried out using Version 2 of the Cochrane risk-of-bias tool for randomized trials (RoB 2) which consists of five domains with assessment results categorized into low risk of bias, there is concern, and high risk of bias. Non-randomized clinical trial studies were assessed using the Risk of Bias In Non-randomized Studies of Interventions (ROBINS-I) which consisted of seven domains with assessment results categorized into low, moderate, serious, critical, and no information risk of bias. Based on the results of the risk of bias assessment, it was found that eight studies had a low risk of bias and one study had a moderate risk of bias. The results of the risk of bias assessment can be seen in (Fig. 2).

### Qualitative synthesis

Qualitative synthesis was carried out on nine studies by extracting important data from each study. The author extracted some data, namely, author’s name, year of publication, number of participants, type of antimicrobial, periodontal clinical parameters, duration of follow-up, and results which are summarized in Table 2.

**Table 2:** Qualitative Synthesis Results

Author (Year)	Number of Participants	Intervention	Periodontal Conditions	Periodontal Clinical Parameters		Follow-up Duration	Result
Reddy et al. (2016)	48	SRP with additional chlorhexidine chips or tetracycline fibers.	There are teeth with a probing periodontal pocket depth of ≥ 5 mm with bleeding on probing or suppuration.		Probing depth, plaque index, and gingival index.	One and three months	All groups showed a significant decrease in mean PI, GI, and PD after one and three months of treatment.
Bankur et al. (2020)	40	SRP with additional chlorhexidine chips or tetracycline fibers.	There are 2 contralateral sides with probing periodontal pocket depth ≥ 5 mm and there is radiographic loss of bone. Clinical attachment loss ≥ 3–5 mm. There is no furcation involvement.		Probing depth, plaque index, and gingival index.	Thirty days	There was no significant difference in the average PI between the two groups. There was a significant difference in the mean of GI and PD with the lower average being achieved by the tetracycline fiber group.
Grover et al. (2014)	20	SRP with added chlorhexidine gel or tetracycline fibers.	Chronic periodontitis with probing periodontal pocket depth of 5–8 mm in molars.		Probing depth and plaque index.	One and three months.	There were no significant differences in the mean PI and PD between the two groups after one and three months of treatment.
Singh et al. (2014)	35	SRP with additional chlorhexidine chips or tetracycline fibers.	There are teeth with probing periodontal pocket depths of 5–8 mm, clinical attachment loss ≥ 3 mm in at least 6 teeth, and bleeding on probing.		Probing depth	One and three months.	Both groups showed a significant decrease in mean PD after one and three months.
Jalaluddin et al. (2022)	60	SRP with the addition of chlorhexidine chips, metronidazole gel, or tetracycline fibers.	Less than 30% of affected teeth had periodontal pockets with a probing depth of ≥ 5 mm and bleeding on probing, and without furcation involvement.		Probing depth, plaque index, and gingival index.	One and three months.	All groups showed significant reductions in mean GI and PD after one and three months. Only the chlorhexidine chip and tetracycline fiber groups showed a significant difference in mean PI after one and three months.
Jhinger et al. (2016)	20	SRP with additional chlorhexidine chips or minocycline microspheres.	Chronic periodontitis with nearly equal probing depths bilaterally (5–8 mm) and showing bleeding on probing.		Probing depth, plaque index, and gingival index.	Three months.	There were no significant differences in the mean PI, GI and PD between the two groups after three months of treatment.
Dheeraj et al. (2020)	60	SRP with additional chlorhexidine chips or tetracycline fibers.	There are at least 2 teeth that are not adjacent to the periodontal pocket with a probing depth of ≥ 5 mm with bleeding on probing.		Probing depth, plaque index, and gingival index.	One and three months.	There was a decrease in mean PI and GI in both groups after one and three months of treatment. There was a significant change in the mean PD after one and three months of treatment.
Abraham et al. (2020)	60	SRP with addition of chlorhexidine gel, metronidazole gel, or tetracycline fibers.	Two or more teeth that are not adjacent and have a periodontal pocket with a probing depth of at least 5 mm and bleeding on probing without furcation involvement.		Probing depth, plaque index, and gingival index.	Thirty days	All groups showed a significant reduction in mean GI and PD after thirty days of treatment. Only the tetracycline group showed a significant difference in mean PI after thirty days of treatment.
Chackartchia et al. (2019)	53	SRP with additional chlorhexidine chips or minocycline microspheres.	There are teeth with a probing pocket depth of ≥ 5 mm.		Probing depth.	Three month	Both groups showed a significant reduction in PD after three months of treatment. There was a significant difference in the reduction of PD between the two groups.

Description: “Scaling and Root Planing” = SRP; “Probing Depth” = PD; “Plaque Index” = PI; “Gingival Index” = IG

### Quantitative synthesis

Quantitative synthesis or meta-analysis was performed on five studies using Mean difference analysis from RevMan 5.4 software with a Confidence Interval (CI) of 95%, then the p-value was determined to determine statistically significant differences. Meta-analyses require information about the mean, standard deviation, and sample size of the existing studies.

#### Quantitative synthesis (Meta-Analysis) between chlorhexidine chips and other antimicrobials

**Fig. 3:**
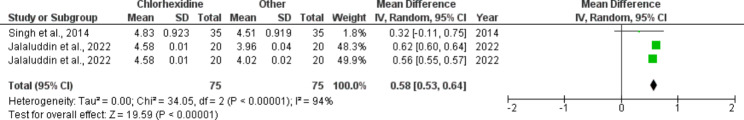
Forest-Plot of Probing Depth after One Month Between Chlorhexidine Chips and Other Antimicrobials

Meta-analysis was conducted to compare the mean probing depth, plaque index, and gingiva after one month of treatment between SPA with chlorhexidine chip addition compared to other local antimicrobials. Meta-analysis between chlorhexidine chips and other local antimicrobials after one month, showed a mean probing depth difference of 0.58 mm (95% CI:[0.53;0.64],p < 0.00001) (Fig. 3).

**Fig. 4:**
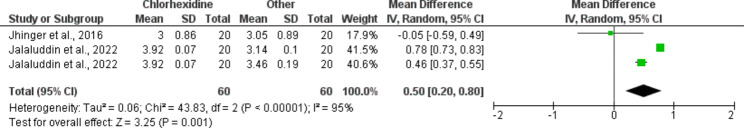
Forest-plot of Probing Depth after Three Months Between Chlorhexidine Chips and Other Antimicrobials

**Fig. 5:**
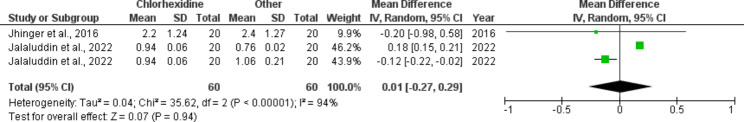
Forest-Plot of Plaque Index after Three Months Between Chlorhexidine Chips and Other Antimicrobials

**Fig. 6:**
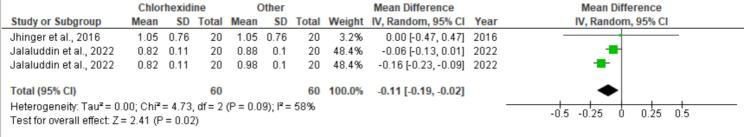
Forest-Plot of Gingival Index after Three Months Between Chlorhexidine Chips and Other Antimicrobials

Quantitative synthesis of probing depth was carried out in three studies, while the plaque and gingival index was carried out in two studies. A meta-analysis was performed to compare the means of probing depth, plaque index, and gingiva after three months of treatment between scaling and root planing with chlorhexidine chip addition compared to other local antimicrobials. From the results of data synthesis after three months between the chlorhexidine chip and other local antimicrobials, there was a difference in probing depth of 0.50 mm (95% CI: [0.20; 0.80], p = 0.001) and more significant results were shown by the use of other antimicrobials (Fig. 4). The mean plaque index difference was 0.01 (95% CI: [-0.27;0.29],p = 0.94) (Fig. 5). In the gingival index there was a mean difference of -0.11 mm (95% CI: [-0.19;-0.02], p = 0.02) and more significant results were shown by the use of chlorhexidine chips(Fig. 6).

#### Quantitative synthesis (Meta-Analysis) between Chlorhexidine Gel and other antimicrobials

**Fig. 7:**
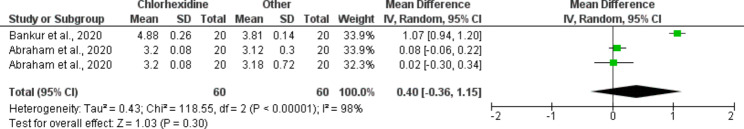
Forest-Plot of Probing Depth after One Month Between Chlorhexidine Gel and Other Antimicrobials

**Fig. 8:**
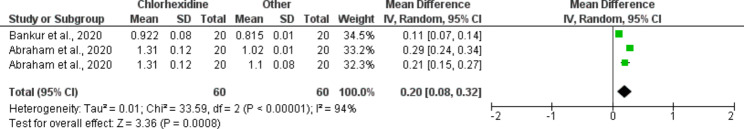
Forest-Plot of Plaque Index after One Month Between Chlorhexidine Gel and Other Antimicrobials

**Fig. 9:**
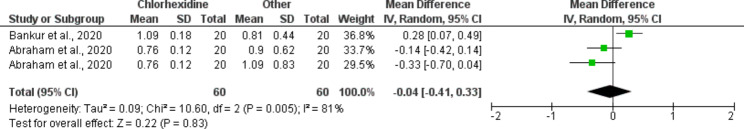
Forest-Plot of Gingival Index after One Month Between Chlorhexidine Gel and Other Antimicrobials

In the results of quantitative synthesis of data between chlorhexidine gel and other local antimicrobials, a difference in the mean probing depth of 0.40 mm (95% CI: [-0.36;1.15], p = 0.30) after one month was found (Fig. 7). The results of the meta-analysis on the mean plaque index showed a mean difference of 0.20 mm (95% CI: [0.08;0.32], p = 0.0008) after one month (Fig. 8). Quantitative synthesis results on the mean gingival index after one month showed a mean difference of -0.04 mm (95% CI: [-0.41;0.33], p = 0.83) (Fig. 9).

## Discussion

Periodontal disease is characterized by inflammation of the supporting tissues of the teeth, mainly caused by plaque and calculus [15, 23]. Scaling and root planing (SRP) is considered the gold standard procedure for non-surgical periodontal therapy [5]. The impact of SRP in some patients or under certain conditions is not optimal therefore additional therapy in the form of antimicrobials has been proposed. ^10,13,^[8] Administration of local antimicrobials after SRP has been shown to be safe and effective and is considered the best approach to treatment of periodontitis [21].

Chlorhexidine is the gold standard for anti-plaque and anti-gingivitis agents [17]. Chlorhexidine as a local antimicrobial for periodontitis treatment consists of 2 forms, namely gel and chip [21]. The addition of xanthan gum to the chlorhexidine gel shows an increase in the viscosity of the chlorhexidine gel therefore the gel can last for at least 2 weeks in the pocket [20]. Chlorhexidine chips were more effective than irrigation or gel without xanthan gum [24]. The slow chip degradation causes the release of chlorhexidine to be gradual and over a longer period of time [15].

Other local antimicrobials that can be used as adjunctive therapy after SRP in periodontitis patients are tetracycline, metronidazole, and minocycline. Tetracyclines are the antibiotics with the highest twenty-four-hour drug release rate. The first study by Goodson et al. which demonstrated in vitro drug release for up to nine days [25]. Metronidazole is a broad-spectrum antibiotic and is active against most periodontal pathogens [26]. Minocycline is one of the most active antibiotics against most of the microorganisms associated with periodontal disease [27]. Minocycline can increase the attachment and spread of fibroblasts which are important for tissue regeneration [28]. The one-month timeframe was chosen because chlorhexidine in gel or chip form was expected to be completely adsorbed after 30 days of placement in the periodontal pocket and because bacteria were expected to return to pre-treatment patterns after three to six weeks of SRP [21, 22]. The three-month timeframe corresponds to the control interval for periodontitis patients [12].

The study conducted by Jalaluddin, et al. showed a lower mean probing depth was found in the tetracycline fiber group followed by metronidazole gel then the chlorhexidine chip group after one month of intervention [29]. Similar results were shown by a study conducted by Singh, et al. that is, lower mean probing depths were found in the tetracycline fiber group than in the chlorhexidine chip group [30]. Different results were shown by studies conducted by Reddy, et al. which showed a lower mean probing depth in patients with additional chlorhexidine chips compared to patients with additional tetracycline fibers [31]. Based on the meta-analysis that has been done, better results have been shown for other local antimicrobial groups (metronidazole and tetracycline).

Jinger, et al. conducted a study which showed that the mean probing depth in the chlorhexidine chip group was lower compared to the minocycline group after three months of intervention [32]. Different results are shown in the results of a study by Jalaluddin, et al. which showed that the mean probing depth of the chlorhexidine chip group was higher than that of the tetracycline fiber group and the metronidazole gel group [29]. Based on the previous meta-analysis, other local antimicrobials showed better results than the chlorhexidine chip group after three months.

Other antimicrobials show better results for several reasons. Tetracycline not only have bactericidal properties and bacteriostatic activity but also have the ability to increase the attachment of fibroblasts to the tooth root surface [22]. Tetracycline has the ability to inhibit collagen breakdown and bone resorption [33]. Metronidazole can reduce the flow of inflammatory cells by inhibiting the production of cytokines IL-1β, IL-6, IL-8, IL-12 and TNF-a so that it can inhibit the destruction of periodontal tissue [34]. The less depth of penetration of the chlorhexidine chip can affect the final result of the treatment [35].

Study conducted by Dheeraj, et al., showed that the plaque index in the group given additional chlorhexidine chips had a lower value than tetracycline fiber after one month [36]. Different results are shown in the results of the study by Reddy, et al. which showed the same plaque index values in the additional groups of chlorhexidine chips and tetracycline fibers [31]. Jalaluddin, et al. conducted a study comparing chlorhexidine chips, tetracycline fibers, and metronidazole gels. The results of the study of Jalaluddin, et al. showed that the plaque index value of the chlorhexidine chip group was lower than the metronidazole gel group but higher than the tetracycline fiber group [29]. There are differences in the writing of the results reported therefore meta-analysis cannot be carried out.

Study conducted by Jhinger, et al. showed that the mean plaque index in the chlorhexidine chip group was lower than that in the minocycline gel group after three months of intervention [32]. The study conducted by Jalaluddin, et al. showed that the tetracycline fiber group produced the lowest mean plaque index after three months of intervention followed by chlorhexidine chip and metronidazole gel [29]. Based on the meta-analysis that has been done, the mean plaque index after three months of treatment showed no significant difference between the chlorhexidine chip group and other local antimicrobial groups (metronidazole, minocycline, and tetracycline). There was no difference in the effectiveness of the meta-analyses which could be due to the small number of samples.

Study by Jalaluddin, et al. showed a lower gingival index value of the chlorhexidine chip group compared to the tetracycline fiber group and the metronidazole gel group after one month [29]. These results are similar to those in the study by Dheeraj, et al.[36] The study by Reddy, et al. showed that the gingival index in the chlorhexidine chip group after one month was higher than the tetracycline fiber group [31]. There are differences in the writing of the results reported therefore meta-analysis cannot be carried out.

Jinger, et al. observed the change in gingival index and found that the chlorhexidine chip group resulted in a greater reduction than the minocycline group [32]. In a study conducted by Jalaluddin, et al. Changes in gingival index were also observed and it was found that the lowest average gingival index was indicated by the chlorhexidine group followed by tetracycline and metronidazole [29]. Based on the meta-analysis, the mean gingival index after three months of treatment showed better results by the additional chlorhexidine chip group. These results can be due to chlorhexidine having the advantage of lasting longer because it can bind to soft and hard tissues intraorally [37]. Tetracycline fibers are reported not to penetrate into the gingiva to a significant distance to kill or suppress tissue invasive organisms, such as *Aggregatibacter actinomycetemcomitans* [38].

Bankur, et al. conducted a comparative study on the effectiveness of chlorhexidine gel and tetracycline fibers in periodontitis patients. The SRP intervention group plus tetracycline fiber showed a lower mean probing depth compared to the chlorhexidine gel group after one month [39]. Similar results were shown by a study by Abraham, et al. which showed a lower mean probing depth in the tetracycline fiber group followed by metronidazole gel and chlorhexidine gel groups [40]. The study by Grover, et al. also showed that the decrease in the mean probing depth after one month was greater in the tetracycline fiber group compared to the chlorhexidine gel group [22]. Based on the meta-analysis that has been done, the mean probing depth after one month of treatment showed no difference in effectiveness between the chlorhexidine adjunct group and the other local antimicrobial adjunct groups (metronidazole and tetracycline). These results can be due to based on Badersten, et al. and Berglundh, et al. maximum results from SRP with additional antimicrobials are expected to occur at three months after treatment [41, 42].

Studies conducted by Abraham, et al. regarding the plaque index comparison, it showed that the tetracycline fiber group had the lowest mean plaque index, followed by the metronidazole gel group, then the chlorhexidine gel [40]. Another study had similar results, namely the study by Bankur, et al. which showed that the tetracycline fiber group had a lower mean plaque index than the chlorhexidine gel group [39]. Based on the meta-analysis that has been done, the mean plaque index after one month of treatment showed a difference in effectiveness between the chlorhexidine gel group and other local antimicrobial groups (metronidazole and tetracycline). Better results have been shown for other local antimicrobial groups (metronidazole and tetracyclines). The decrease in plaque index in the tetracycline group occurred due to control of subgingival plaque which then had the effect of inhibiting the development of supragingival plaque [43]. Baker, et al. showed that tetracycline has the ability to adsorb on saliva-coated email so that it can inhibit plaque formation [44].

Study by Abraham, et al. showed the mean gingival index after one month of intervention in the chlorhexidine gel group showed the lowest average followed by tetracycline fiber then metronidazole gel [40]. Studies that have different results, namely the study by Bankur, et al. showed that the tetracycline fiber group had a lower average gingival index than the chlorhexidine gel group [39]. Based on a meta-analysis, the mean gingival index after one month of treatment showed no difference in effectiveness between the chlorhexidine gel group and other local antimicrobial groups (metronidazole and tetracycline). These results can be due to based on Badersten, et al. and Berglundh, et al. maximum results from SRP with additional antimicrobials are expected to occur at three months after treatment [41, 42].

The limitation of this study was that meta-analysis of the gingival and plaque indices after SRP with chlorhexidine or other local antimicrobials was not possible due to the lack of a uniform number of journals. Unable meta-analysis and different study results make it difficult to draw conclusions. The limited number of journals also makes it impossible to conduct subgroup research between local antimicrobial types. Possible adverse/adverse effects of subgingival insertion such as allergy, pain, discomfort, and periodontal abscess formation were not evaluated.

## Conclusion

The average probing depth in the chlorhexidine chip group after one month showed fewer effective results compared to metronidazole and tetracycline. The average probing depth in the chlorhexidine chip group after three months showed fewer effective results compared to the minocycline, metronidazole, and tetracycline groups. The plaque index in the chlorhexidine chip group after three months showed no difference in effectiveness compared to the minocycline, metronidazole, and tetracycline groups. The gingival index in the chlorhexidine chip group after three months showed more effective results compared to the minocycline, metronidazole, and tetracycline groups. The mean probing depth and gingival index in the chlorhexidine gel group after one month showed no difference in effectiveness compared to the metronidazole and tetracycline groups. The plaque index in the chlorhexidine gel group after one month was less effective than the metronidazole and tetracycline groups.

## Data Availability

The datasets used and/or analysed during the current study available from the corresponding author on reasonable request.
